# The Functional Interaction of KATP and BK Channels with Aquaporin-4 in the U87 Glioblastoma Cell

**DOI:** 10.3390/biomedicines12081891

**Published:** 2024-08-19

**Authors:** Fatima Maqoud, Laura Simone, Domenico Tricarico, Giulia Maria Camerino, Marina Antonacci, Grazia Paola Nicchia

**Affiliations:** 1 Section of Pharmacology, Department of Pharmacy-Pharmaceutical Sciences, University of Bari “Aldo Moro”, 70125 Bari, Italy; f.maqoud@gmail.com (F.M.); giuliamaria.camerino@uniba.it (G.M.C.); marina.antonacci@uniba.it (M.A.); 2 Functional Gastrointestinal Disorders Research Group, National Institute of Gastroenterology Saverio de Bellis, I.R.C.C.S. Research Hospital, 70013 Castellana Grotte, Italy; 3 Cancer Stem Cells Unit, Fondazione IRCCS Casa Sollievo della Sofferenza, Viale Cappuccini, snc, 71013 San Giovanni Rotondo, Italy; l.simone@operapadrepio.it; 4 Department of Biosciences, Biotechnologies and Environment, University of Bari Aldo Moro, 70121 Bari, Italy

**Keywords:** glioblastoma, BK channels, KATP channels, aquaporin-4, M1-AQP4 (AQP4-tetramers forming isoform), M23-AQP4 (AQP4-OAPs forming isoform), cell cycle

## Abstract

K^+^ channels do play a role in cell shape changes observed during cell proliferation and apoptosis. Research suggested that the dynamics of the aggregation of Aquaporin-4 (AQP4) into AQP4-OAP isoforms can trigger cell shape changes in malignant glioma cells. Here, we investigated the relationship between AQP4 and some K^+^ channels in the malignant glioma U87 line. The U87 cells transfected with the human M1-AQP4 and M23-AQP4 isoforms were investigated for morphology, the gene expression of *KCNJ8*, *KCNJ11*, *ABCC8*, *ABCC9*, *KCNMA1*, and Cyclin genes by RT-PCR, recording the whole-cell K^+^ ion currents by patch-clamp experiments. AQP4 aggregation into OAPs increases the plasma membrane functional expression of the Kir6.2 and SUR2 subunits of the KATP channels and of the *KCNMA1* of the BK channels in U87 cells leading to a large increase in inward and outward K^+^ ion currents. These changes were associated with changes in morphology, with a decrease in cell volume in the U87 cells and an increase in the ER density. These U87 cells accumulate in the mitotic and G2 cell cycle. The KATP channel blocker zoledronic acid reduced cell proliferation in both M23 AQP4-OAP and M1 AQP4-tetramer-transfected cells, leading to early and late apoptosis, respectively. The BK channel sustains the efflux of K^+^ ions associated with the M23 AQP4-OAP expression in the U87 cells, but it is downregulated in the M1 AQP4-tetramer cells. The KATP channels are effective in the M1 AQP4-tetramer and M23 AQP4-OAP cells. Zoledronic acid can be effective in targeting pathogenic M1 AQP4-tetramer cell phenotypes inhibiting KATP channels and inducing early apoptosis.

## 1. Introduction

To date, several types of brain tumors are known, and they differ considerably in outcome. According to their malignancy, they are classified from grade I to grade IV. Glioblastoma multiforme (GBM), one of the most common adult primary brain tumors arising from glial cells [1,2], exhibits the highest malignancy (grade IV) and median survival of approximately 15 months [3]. The management of glioblastomas poses a clinical challenge due to the ability of glioma cells to rapidly infiltrate healthy brain parenchyma, allowing the cancer to escape control through surgical resections and localized radiation therapy and promoting recurrence in other brain regions [4]. Great attention is given to the importance of cerebral fluid homeostasis, as its alteration is a common feature of brain tumors [3,5]. In the last 15 years, a growing body of evidence has underscored the significance of the splicing, dysregulated expression, and altered function of ion channels and aquaporins (AQPs) in various cancer types. These molecular aberrations play crucial roles in multiple cancer processes, highlighting their potential as important therapeutic targets and biomarkers for cancer diagnosis and treatment [6]. Their pivotal role in determining the fate of these cells has led to the characterization of certain ion channels as oncogenic channels due to their impact on cancer development and/or progression [7]. Numerous studies conducted across various laboratories have consistently demonstrated that the manipulation of ion channels and/or AQPs can effectively impede the growth and migration of cancer cells. Investigations have reported the successful utilization of substances targeting different classes of AQPs and ion channels, such as the Ca^2+^-activated K^+^ channels of large (BK) and intermediate conductance (IKCa), and ATP-sensitive potassium channels (KATP) to inhibit cell migration, invasion, and metastasis in vitro and in vivo for diverse types of carcinomas [4,8,9]. 

To date, researchers have identified a minimum of 13 distinct isoforms of AQPs. These specialized channels are extensively expressed in diverse epithelial and endothelial cells within mammals and play a crucial role in modulating cell capacity in volume control and response to an environmental osmotic change [10,11]. The most abundant AQP in the CNS is the glial water channel AQP4 expressed in astrocytes. Astrocytes exhibit a distinct polarized distribution of AQP4, with a prominent presence in the astrocytic feet that come into contact with blood vessels at the blood–brain barrier (BBB) and in those forming the glial limitans [12]. In these sites, under normal conditions, AQP4 forms heterotetramers in the plasma membrane consisting mainly of the two different isoforms AQP4-OAP and AQP4-tetramer. A smaller amount of the recently discovered M23-AQP4 and M1-AQP4 is also expressed and shown to be important to lock AQP4 at astrocyte processes. The isoform ratio controls the aggregation of AQP4 into supramolecular structures called orthogonal particle arrays (AQP4-OAPs) [13]. Interaction with intracellular scaffold proteins, such as α-syntrophin, determines the formation and localization of OAPs in astrocytes [14,15,16]. It is interesting to note that these complexes also contain the inwardly rectifying potassium channel such as the ATP-sensitive K^+^ channel (KATP), and the large conductance calcium-activated K^+^ channels (BK); in particular, the K^+^ ions are absorbed by the astrocytes, and the water follows them osmotically through the AQP4 water channels to regulate the cell volume [17]. A pivotal player in preserving water and ion balance within the central nervous system, AQP4 has garnered attention for its vital role in brain edemas. 

Recently, researchers have uncovered its additional involvement in cell migration, further expanding its repertoire of functions. Notably, an increased expression of AQP4 has been observed in glioblastoma multiforme (GBM) [18,19,20]. Simone and coworkers [13,21] demonstrated that the aggregation of AQP4 in OAPs influences the biology and the fate of glioma cells. In particular, M23 AQP4-OAPs triggered cell shape changes associated with alterations in the cytoskeleton of F-actin, leading to glioma cell apoptosis. Conversely, the expression of M1 AQP4-tetramers that are unable to aggregate into OAPs increased the invasiveness, cell migration, and metalloproteinase-9 activity of glioma cells escaping apoptosis [13].

K^+^ ion channels regulating cell volume and resting potentials play a role in cancer cell progression mechanisms [22,23,24]. For example, the overexpression of different potassium channel subunits, composing the BK and KATP channels, has been found in tumor tissues of different origins and especially in those of gliomas and other brain tumors (Table 1). Conducting an extensive search using PubMed on glioma/brain-cancer-related articles of *ABCC8*, *ABCC9*, *KCNJ11*, *KCNJ8*, *AQP4*, and *KCNMA1* genes has yielded interesting results. Apart from the well-established roles of AQP4 and K^+^ ion channels in diverse pathological conditions, the genes responsible for encoding AQPs and K^+^ ion channels exhibit differential expressions and mutations in different cancer types. This discovery highlights the potential significance of these genes in the context of cancer development and may offer valuable insights into their involvement in cancer-related processes [25] (Table 1).

The hypothesis that there is a relationship between the overexpression of the K^+^ channel and the generation and growth of malignant tumors has been confirmed in different studies as these channels are involved in cell proliferation, apoptosis, and differentiation [26,39,40].

Drugs that specifically block K^+^ ion channels have shown anticancer effects by directly inhibiting tumor growth or by improving the efficacy of chemotherapy or cytotoxic drugs as a combined therapeutic strategy [22]. For instance, small-molecule KATP channel inhibitors like glibenclamide, which reduced cell proliferation in a variety of cells, including leiomyoma and MDA-MB-231 cancer cells, were extensively investigated in cancer. More recently, a monoclonal antibody (Y4) targeting the cap domain of TASK-3 has garnered attention for its potential in inhibiting the growth of human lung cancer xenografts and reducing breast cancer metastasis in mice [22]. The first polyclonal antibody (BIL010t; Biocentre) targeting a non-functional form of P2X7 (nfP2X7) has reached clinical trials for the treatment of basal cell carcinoma [23]. In addition, short peptides derived from venom are under investigation. On the other hand, several studies have shown the impact of Ca^2+^-activated potassium channels (BK) on tumor cell proliferation and their association with the tumorigenesis process in patient and animal models [22,40].

In this study, we investigated the functional/expression changes of KATP and BK channel subunits by patch-clamp and RT-PCR experiments in U87 cells transfected with AQP4 isoforms, enabling different aggregation states (AQP4 OAPs vs. AQP4 tetramers) compared to the non-transfected cells (WT). The aim was to evaluate the relationship between the KATP and BK channels’ functional expression and the role of the AQP4 aggregation state in U87 cells.

KATP channel inhibitors like sulfonylurea drugs are known to induce apoptosis in different cell lines and tissues [41] with antiproliferative effects. The BK channel openers also induce apoptosis [42]. Given the expected role of the KATP and BK channels in glioblastomas, we also investigated the antiproliferative effects of zoledronic acid, a recently identified KATP channel blocker targeting either the Kir6.1/2 and SUR2 subunits in native cells [43,44] and in cell lines transfected with KATP channel subunits [45]. This drug is capable of activating BK channels in MD-MBA-231 breast cell lines with apoptosis [46], and it is a modulator of the TRPV1 channel [47]. It is also used in the treatment of osteoporosis, bone metastasis, and myeloma, increasing the overall survival of patients with malignancy [48,49].

## 2. Materials and Methods

### 2.1. Cell Lines

The U87 MG (ATCC HTB-14) cell line, derived from a malignant glioma in a female patient using the explant technique, was procured and authenticated from the ATCC (Manassas, VI, USA, www.lgcstandards-atcc.org) [50]. Experiments were conducted using cells from passages 1 to 20. Routine mycoplasma testing was carried out, either using MycoAlert substrate (Lonza Group AG, Basel, Switzerland, https://bioscience.lonza.com) or fluorescence staining with DAPI. The cells were cultured in DMEM/F12 supplemented with 10% FBS and 100 units of penicillin/streptomycin in a 5% CO_2_ environment at 37 °C. To maintain exponential growth, the cells were subcultured every 3 days and maintained at approximately 80% confluency until further experiments were conducted. Cell culture reagents were obtained from Euro Clone (Pero, Italy, www.euroclonegroup.it).

### 2.2. Constructs and Transfection

Human M1M23I-AQP4 (referred to as AQP4-tetramers) and M23-AQP4 (referred to as AQP4-OAPs) coding sequences were cloned into pTarget vectors (A1410, Promega, Madison, WI, USA, www.promega.com). For the experiments, the mutated form of M1-AQP4 (M23I) was used, which has been previously characterized and shown to exclusively produce AQP4-tetramers (PMID: 20007705). To prepare for transfection, cells were seeded in the antibiotic-free medium when they reached 70% confluence, 24 h before the actual transfection. Lipofectamine 3000 (L3000015, Thermo Fisher, Waltham, MA, USA, www.thermofisher.com) was employed for the transient transfection, following the manufacturer’s protocol, in the OptiMEM growth medium. After 24 h of transfection, recordings of the potassium currents and the immunofluorescence analysis were performed [21].

### 2.3. Drugs and Solutions

K^+^ channel modulators glyburide/glibenclamide (GLIB) cat. N° PHR1287, TEA, diazoxide (DIAZO), iberiotoxin (IbTX) cat. N° I5904, and BaCl_2_ cat. N° 449644 were purchased from Sigma (SIGMA Chemical Co., Milan, Italy). Zoledronic acid was prepared in our labs [45]. DMSO was observed to have no impact on the channel currents or cell viability [51].

### 2.4. Antibodies

For immunofluorescence analysis, the following antibodies were employed: rabbit polyclonal anti-AQP4 (H-80) (scbt, sc-20812, RRID: AB_2274338) at a dilution of 1:500 and Alexa-Fluor 594-conjugated donkey anti-rabbit (Thermo Fisher, A21207, RRID: AB_141637) at a dilution of 1:1000. To stain F-Actin, conjugated Phalloidin Alexa Fluor 488 was used at a dilution of 1:500 (A12379, www.thermofisher.com).

### 2.5. Nuclear Staining

U87 cells were plated in a 96-well plate with a density of 3 × 103 cells per well and exposed to either zoledronic acid or diazoxide for 18 h. After the incubation, the cells were fixed and processed for immunofluorescence as previously described. Alternatively, some cells were treated with 10 μg/mL of DAPI for 30 min at 37 °C to stain the cell nuclei. Following the staining procedures, the cells were examined under an inverted fluorescence microscope. The nuclei were automatically detected and analyzed for their mean nuclear area using Fiji software (Fiji, RRID:SCR_002285, Fiji Dresden, Germany). For each experimental condition, nuclei from at least three fields were analyzed, and the experiments were independently repeated three times.

### 2.6. Immunofluorescence and Quantitative Analysis

U87 cells were fixed using 4% paraformaldehyde (PFA) for 15 min and then washed three times with PBS. To facilitate permeabilization, 0.3% Triton X-100 was applied. Following this, a 30 min blocking step with 0.1% bovine albumin serum (BSA) was carried out at room temperature. Next, the cells were subjected to a 1 h incubation with primary antibodies, followed by PBS washes containing BSA. For secondary antibody labeling, Alexa-Fluor-conjugated secondary antibodies were used. Finally, the cells were mounted using a medium containing 50% glycerol and 1% n-propyl gallate in PBS, along with DAPI for nuclear staining. To visualize the immunostained cells, an epifluorescence photomicroscope equipped with 16× and 40× oil PL FL FLUOTAR objectives from Leica Microsystems (Wetzlar, Germany, www.leica-microsystems.com) was utilized. Digital images were captured using a DMX1200 camera from Nikon (Tokyo, Japan, www.Nikon.it) and processed using LAS AF software, 4.0 provided by Leica Microsystems GmbH (RRID:SCR_013673). The auto contrast function in Photoshop CS5 (RRID:SCR_014199) was applied to the entire images for image enhancement. For quantitative analysis following immunofluorescence, three to five different fields were chosen from each of the three independent experiments conducted on separate days. Fiji software (Fiji, RRID:SCR_002285) was used to accurately count the cells within each field, and the data were analyzed with GraphPad Prism 9 software (GraphPad Prism, RRID:SCR_002798).

### 2.7. Morphological Analysis

Morphological analysis was carried out to assess the presence of two cell types: round-shaped cells and irregular-shaped cells exhibiting a star-like phenotype. The cells displaying these altered morphologies were distinctly identifiable from other cells in each field. Two independent investigators performed the analysis in a blinded manner. The quantification of cells with altered morphology was accomplished using Fiji software (Fiji, RRID:SCR_002285), and the data were analyzed using GraphPad Prism 9 software (GraphPad Prism, RRID:SCR_002798).

### 2.8. Cell Viability Assay

To examine the effect of K^+^ channel modulators on cell proliferation, cell viability was assessed using the 3-(4,5-dimethylthiazol-2-yl)-2,5-diphenyltetrazolium bromide (MTT) test. This assay is based on the principle that the reduction of MTT tetrazolium salt is primarily carried out by mitochondrial dehydrogenases. Following a 24 h exposure or after specific incubation periods in the culture medium, an MTT stock solution (5 mg/mL in medium) was added to each dish at a volume equivalent to one-tenth of the original culture volume. The cells were then incubated for 2 h at 37 °C in a humidified CO_2_ environment. Subsequently, the supernatant was removed, and the cells were dissolved in 150 μL of DMSO–ethanol in the ratio 1:1., which was used to solubilize the formazan crystals formed as a result of the MTT reduction. Formazan formation was quantified using spectrophotometry, measuring the absorbance at 570 nm. The amount of formazan produced is proportional to the number of viable cells in the culture, allowing for the evaluation of cell viability under different experimental conditions.

### 2.9. Patch-Clamp Experiments

The whole-cell patch-clamp experiments for recorded membrane currents were performed in asymmetrical K^+^ ion concentration in physio-logical conditions using pipettes with the resistance of 3–5 MΩ. The pipette solution contained 132 mM KCl (P3911, Sigma Chemical Co., Milan, Italy), 1 mM ethylene glycol-bis (β-aminoethyl ether)-N, N, N′, N′-tetra acetic acid EGTA (E3889, Sigma Chemical Co., Milan, Italy), 10 mM NaCl (S9888, Sigma Chemical Co., Milan, Italy), 2 mM MgCl_2_ (M8266, Sigma Chemical Co., Milan, Italy), 10 mM HEPES (H3375, Sigma Chemical Co., Milan, Italy), 1 mM Na_2_ATP (A26209, Sigma Chemical Co., Milan, Italy), and 0.3 mM Na_2_GDP (51060, Sigma Chemical Co., Milan, Italy) (pH = 7.2). The bath solution contained 142 mM NaCl (S9888, Sigma Chemical Co., Milan, Italy), 2.8 mM KCl (P3911, Sigma Chemical Co., Milan, Italy), 1 mM CaCl_2_ (C8106, Sigma Chemical Co., Milan, Italy), 1 mM MgCl_2_ (M8266, Sigma Chemical Co., Milan, Italy), 11 mM glucose (D9434, Sigma Chemical Co., Milan, Italy), and 10 mM HEPES (H3375, Sigma Chemical Co., Milan, Italy) (pH = 7.4). CaCl_2_ was added to the pipette solutions to give a free Ca^2+^ ion concentration of 1.6 × 10^−6^ M in whole-cell experiments. The calculation of the free Ca^2+^ ion concentration in the pipette was performed using MaxChelator software, 1 (Stanford University, Stanford, CA, USA). Drug actions on the K^+^ ion currents recorded during instantaneous I/V relationships were investigated by applying a depolarization protocol in response to voltage pulses from −120 mV to +120 mV (Vm) in 20 mV steps. Currents were expressed as densities (pA/pF) to control cell size/capacitance differences. All experiments were performed as described in Scala et al. [52].

### 2.10. Polymerase Chain Reaction

Total RNA was isolated and purified from the U87 cells WT and U87 transfected by AQP4-OAP or AQP4-tetramer with Trizol reagent (Invitrogen, Thermo Fisher Scientific Inc., Waltham, MA, USA) and quantified using a spectrophotometer (ND-1000 Nano-Drop, Thermo Fisher Scientific Inc., Waltham, MA, USA). Real-time PCR was performed in triplicate using the Applied Biosystems Real-time PCR 7500 Fast system (Waltham, MA, USA). The mRNA expression of the genes was normalized to the best housekeeping gene β-actin (Actb) selected from glyceraldehyde-3-phosphate dehydrogenase (Gapdh), using Best Keeper 1 (Tum School of Life Science, München, Germany) and NorFinder 1 (Brendstrupgardsvej 21A, 8200, Aerhus University, Denmark) softwares. TaqMan hydrolysis primer and probe gene expression assays were obtained from Life Technologies (Carlsbad, CA, USA) with the following assay *KCNMA1* ID: Hs01119504_m1; *KCNJ11* ID: Hs00265026_s1; *KCNJ8* ID: Hs00958961_m1; *ABCC8* ID: Hs01093752_m1; *ABCC9* ID: Hs00245832_m1; *TRPV1* ID: Hs00218912_m1; *Cyclin A2* I; D: Hs00996788_m1; *Cyclin B1* ID: Hs01030099_m1; *Cyclin E* ID: Hs01026536_m1; *B-actin*: ID: Hs01101944_s1. All gene expression experiments were conducted following the MIQE guidelines [53,54,55].

### 2.11. Statistical Analysis

Data were collected and analyzed using various software tools, including Excel software Microsoft 365 (Microsoft Corporation One Microsoft Way Redmond, WA, USA, RRID: SCR_016137), Clampfit 10.5 (Molecular Devices, RRID: SCR_011323), and SigmaPlot 10.0 (Systat Software, RRID: SCR_003210). The results are expressed as mean ± SEM unless stated otherwise. The number of replicates for each experimental dataset is mentioned in the figure description. For statistical analysis, one-way analysis of variance (ANOVA) followed by multiple comparison tests was applied, with a significance level of *p* < 0.05, unless otherwise specified. Additionally, the Student *t*-test was used to compare the significance between means. Statistical significance was considered when *p* < 0.05 unless explicitly stated otherwise.

## 3. Results

### 3.1. AQP4-OAP Expression in U87 Cells

**Figure 1:**
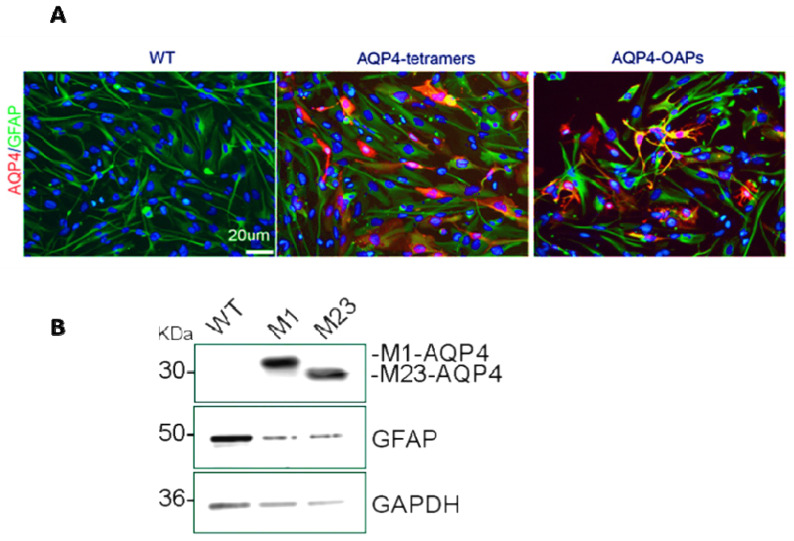
Expression of AQP4 in U87 cells. (**A**) Epifluorescence images of U87wt and U87 expressing M1 AQP4-tetramers or M23 AQP4-OAPs. AQP4 staining is shown in red and DAPI in blue. Scale bar 20 μm. (**B**) Immunoblot detection of AQP4 expression levels in U87 cells transfected with M1-AQP4 (AQP4-tetramers) and M23-AQP4 (AQP4-OAPs). GFAP and GAPDH were used to normalize for equal loading.

After transfecting *AQP* isoforms into U87 cells, we routinely conducted immunofluorescence assays on both the wild-type (WT) cells and the transfected cells to confirm the success of the experiment (Figure 1). Moreover, our knowledge about *AQP4* expression in glioma cells is currently limited. In a study by McCoy et al. [56], the expression of *AQP4* was investigated in various commonly used human glioma cell lines (D54, D65, STTG1, U87, and U251) and several acute patient biopsies using PCR, Western blot, and immunocytochemistry. These findings were compared with non-malignant astrocytes and normal brain tissues. The researchers observed that all glioma patient biopsies expressed AQP4. However, when these cells were isolated and cultured as cell lines, they lost AQP4 protein expression. This phenomenon poses challenges in studying the endogenous expression of AQP4 in cultured cells and utilizing known inhibitors of AQP4, such as TGN-020. This issue has been commonly reported in studies involving glioma cell lines. In our previous research, we also found negative staining for AQP4 in U87 and U251 cell lines, as well as in GL95 primary cell cultures [13].

### 3.2. Whole-Cell Inward and Outward Macroscopic K^+^ Currents Recorded in U87wt Cells and Effects of the K^+^ Channel Modulators on Cell Proliferation

**Figure 2:**
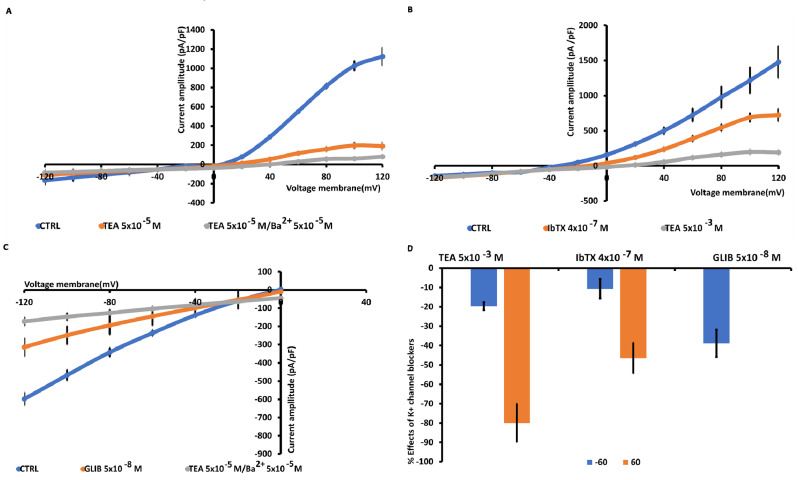
Characterization of inward and outward macroscopic K^+^ ion currents recorded in U87wt cells. The currents were recorded using a whole cell configuration under physiological concentration of K^+^ ions in the bath and pipette and were obtained in response to voltage pulses from −120 to +120 mV in increments of 20 mV, starting at HP = −60 mV (Vm) and intracellular-free Ca^2+^ ions (1.6 × 10^−6^ M). (**A**) TEA (5 × 10^−3^ M) suppressed the outward K^+^ ion currents, and TEA/Ba^2+^ (5 × 10^−3^ M) fully suppressed also the inward K^+^ ion currents. (**B**) The selective BK channel blocker IbTX (4 × 10^−7^ M) reduced the outward K^+^ ion currents that were fully reduced by TEA (5 × 10^−3^ M). (**C**) KATP currents recorded in U87wt cells in low intracellular ATP 1 × 10^−3^ M. Glibenclamide (5 × 10^−8^ M) suppressed the inward currents, suggesting the presence of KATP channels in the cells. Cells of the same size were selected for patch-clamp experiments. Each point represented the mean ± SEM (N patches = 10^–15^). (**D**) Percentage of blocking of K^+^ ion currents in the presence of the antagonists with respect to the control condition at 60 mV and −60 mV (Vm).

**Figure 3:**
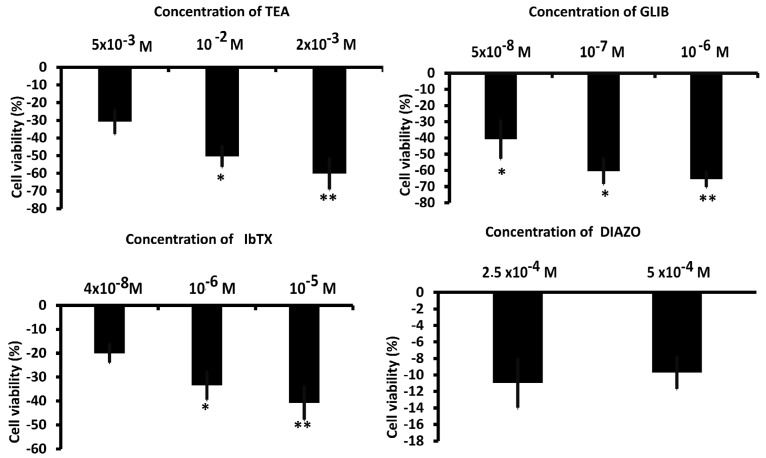
Effects of antagonists and agonists of the K^+^ channels on the proliferation of U87wt cells. * *p* < 0.05 and ** *p* < 0.01 when compared to the experimental group not treated after 48 h of incubation. Each point represented the mean ± SEM.

Large outward K^+^ currents were recorded in U87 cells (Figure 2A). To perform an electrophysiological characterization and identify K^+^ channels that are present endogenously in U87 cells and that may influence U87 cell behavior, including proliferation, we evaluated the effects of a variety of K^+^ channel blockers, and an MTT assay was used to determine cell viability after drug treatment. The effects of TEA, a non-specific outward transient and voltage-dependent K^+^ channel blocker, of the glibenclamide (GLIB), a specific ATP K^+^ channel blocker, and the specific BK-channel blocker iberiotoxin (IbTX) were investigated. As shown in Figure 2B,D, at 60 mV, TEA and IbTX reduced the current by approximately 80% and 46%, respectively. Additionally, GLIB, under conditions of low ATP content (1 mM) in the pipette and at a membrane voltage of −60 mV, decreased the inward current by about 40% (Figure 2C,D). Regarding the effects on cell viability detected at 48 h, as shown in Figure 3, TEA, GLIB, and IbTX reduced the number of U87 cells compared to the untreated control condition. However, the KATP channel agonist diazoxide (DIAZO) had no significant effect on cell proliferation. TEA, GLIB, and IbTX, at the highest concentrations, reduced cell viability by approximately −60.14%, −65.4 %, and −40.77 %, respectively. In our case, high concentrations of these drugs were used especially for IbTX (Figure 3). This can be due to the fact that IbTX is relatively impermeant and do not act on the mitochondrial BK channel. The data suggest that the large outward and inward currents mostly sustained by the BK channels and KATP channels, respectively, contributed to the cell proliferation in this cell. A contribution of other ion channels to the cell currents and proliferations cannot be excluded. For example, the TRPV1 channel is expressed in these cells and may contribute to outward currents to a minor extent, as all outward currents were inhibited by the non-selective Kv/BK channel blocker TEA.

### 3.3. AQP4 Aggregation State Affects the TEA-K^+^ Sensitive Currents in U87 Glioma Cells

**Figure 4:**
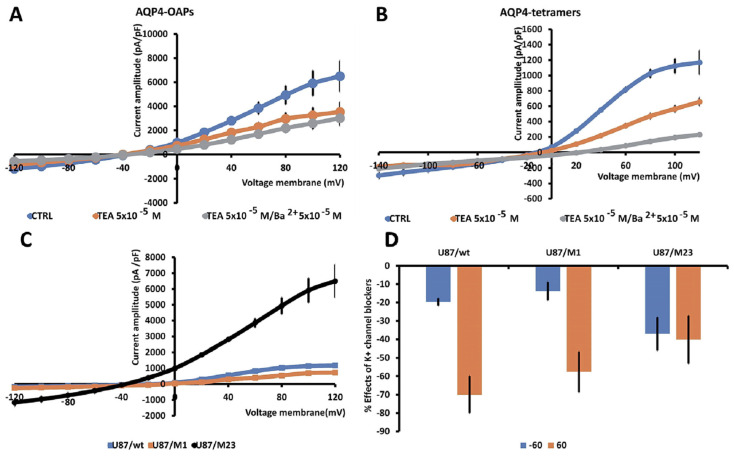
Characterization of macroscopic inward and outward K^+^ ion currents recorded in U87wt cells and after transfection with M1 AQP4-tetramer or M23 AQP4-OAP. Whole cell currents were recorded under physiological concentration of K^+^ ions in the bath and pipette and were obtained in response to voltage pulses from −120 to +120 mV in 20 mV increments, starting at HP = −60 mV (Vm). (**A**) Macroscopic K^+^ ion currents recorded in AQP4-OAP-transfected U87 cells. The presence of AQP4-OAPs caused a significant increase in the inward and outward currents that were reduced by TEA and BaCl_2_. (**B**) Macroscopic K^+^ ion currents recorded in AQP4-tetramer-transfected U87 cells. The presence of M1 AQP4-tetramers caused a significant increase in the inward currents; however, the outward K^+^ ion currents were reduced in the amplitude. (**C**) The presence of M23 AQP4-OAPs caused a large increase in the currents at negative and positive membrane potentials vs. not transfected cell. Instead, the presence of AQP4-tetramers led to an increase in the currents at negative membrane potentials; conversely, the outward K^+^ ion currents decreased at positive membrane potentials. Data were pooled from N patches = 10–12. (**D**) Percentage of reduction in the K^+^ ion currents in the presence of the antagonist TEA with respect to the control condition at 60 mV and −60 mV (Vm). Each point represents the mean ± SEM.

The transfection of U87 cells with the tetramer forming AQP4-tetramer or the OAP forming AQP4-OAP isoform allowed us to monitor the electrophysiological behavior of these cells, creating a system to study the impact of AQP4-aggregated (M23 AQP4-OAPs) and disaggregated pathogenic (M1 AQP4-tetramers) forms on electrical activity. The presence of AQP4 aggregation in AQP4-OAPs leads to a significant increase in both inward and outward K^+^ ion currents, with an increase of about 370% at −60 mV (Vm) and of 400% at 60 mV (Vm), respectively, vs. U87 cells not transfected (Figure 4A). Instead, the aggregation of AQP4-tetramers causes a significant increase in the inward K^+^ ion currents of about 76% at −60 mV, while the outward currents suffered a decrease of −52% at 60 mV vs. U87 cells not transfected (Figure 4B,C). After transfection, the currents were responsive to the unselective blockers TEA and BaCl_2_ (Figure 4A,B,D). In U87/M23 cells, the TEA sensitivity was observed at negative voltages where the BK should not be activated; the γ subunit may account for this effect in neurons [57]. BK channel phosphorylation may account for BK channel openings increasing TEA-sensitive channel currents at negative membrane potentials. In addition, the openings of the intermediated calcium-activated K^+^ channel (IKCa), which is active at more negative potentials, contribute to the currents in our cells. This has been observed in U87-M GBM cells [58] where the BK and IKCa open in response to the hypotonic-induced activation of mechanosensitive channels with an influx of Ca^2+^ ions. Moreover, in line with the large K^+^ ion currents recorded in the AQP4-OAPs, these cells were more repolarized vs. the other cells with a resting potential, respectively, of −40 mV ± 12 and −10 ± 5 mV in the AQP4-OAP and AQP4-tetramer-transfected U87 cells and −10 ± 7 mV in the U87 cells that were not transfected.

### 3.4. AQP4 Aggregation State Changes the Expression Profile of KCNMA1, KCNJ11, ABCC8, and ABCC9 Genes in U87 Glioma Cells

**Figure 5:**
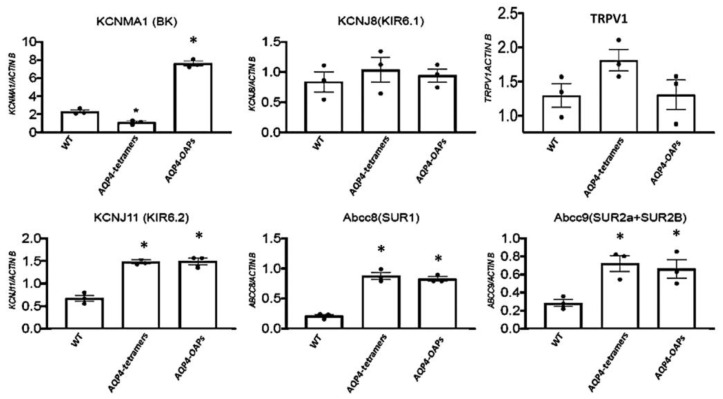
Expression profile of *KCNMA1*, *KCNJ11*, *KCNJ8*, *ABCC8*, *ABCC9*, and *TRPV1* genes in U87 glioma cells in the presence of the malignant M1 AQP4-tetramer and M23 AQP4-OAP aggregation vs. wt condition. * *p* < 0.05 when compared to the experimental group not treated (WT) after 48 h of incubation. Each point represented the mean ± SEM.

The AQP4 aggregation state similarly and significantly caused an upregulation of the *KCNJ11*, *ABCC8*, and *ABCC9* genes in U87 glioma cells (Figure 5). A different behavior concerns the *KCNMA1* with AQP4 tetramers, leading to a significant downregulation in line with the reduced outward current observed in these cells. On the contrary, AQP4-OAPs caused a statistically significant upregulation of the *KCNMA1* in U87 glioma cells in line with the elevated outward currents recorded in these cells. A not statistical enhancement of the expression of *TRPV1* gene was found in the pathogenic AQP4-tetramers (Figure 5).

### 3.5. AQP4 and Kir6.2 Are Involved in Glioma Apoptotic Fate

**Figure 6:**
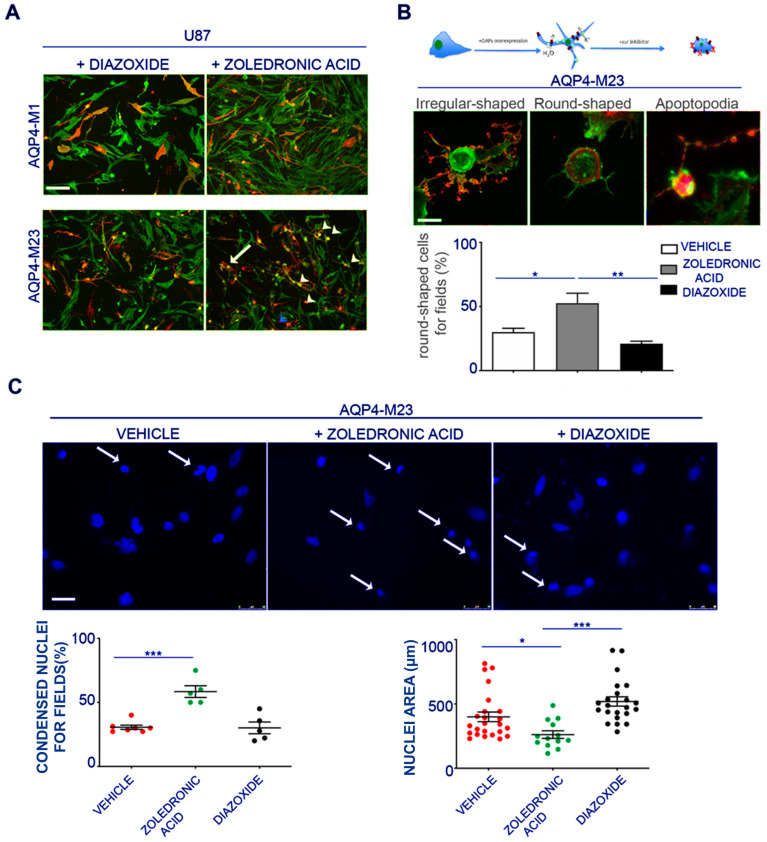
Effect of pharmacological inhibition of the Kir6.2-SUR2 channel activity with zoledronic acid on AQP4s expressing U87 cells. (**A**) Epifluorescence images of U87 cells expressing AQP4-tetramers or AQP4-OAPs treated with diazoxide or zoledronic acid. AQP4 staining is shown in red, and DAPI for nuclear staining is in blue. Phalloidin (in green) was used to visualize F-actin. The white arrowheads indicate the round-shaped cells, the arrows indicate irregular-shaped cells, and the blue arrowheads indicate the apoptotic beads. Scale bar 100 μm. ((**B**) top) Drawing/diagram showing the morphological change of U87 cells expressing AQP4-OAPs after pharmacological treatment with zoledronic acid according Coffin hypothesis and relative epifluorescence images. ((**B**) bottom) Epifluorescence images of U87 expressing AQP4-OAPs treated with zoledronic, showing the irregular-shaped cell, the round-shaped cell, and the apoptosis. AQP4 staining is shown in red. Phalloidin (in green) was used to visualize F-actin. Scale bar 10 µm. A histogram was created to display the percentage of round-shaped cells per field in U87 cells and those expressing AQP4-OAPs after treatment under different conditions. The conditions include treatment with zoledronic acid (an inhibitor), control conditions (vehicle), and diazoxide (an agonist). The histogram compares these percentages across the different treatment groups, highlighting the effects of the inhibitor and agonist on cell morphology. Values are expressed as mean ± SEM of percentage of cells with altered cell morphology out of the total number of transfected cells per field. ** *p* < 0.005; ((**C**) top) Representative image of expressing AQP4-OAPs in control condition or after treatment with zoledronic or diazoxide as indicated and stained with DAPI to visualize nuclei. Scale bar 50 µm. ((**C**) bottom) Dot plot showing the analysis of % of condensed nuclei for field and the nuclear area of images in A. Values are expressed in µm^2^ and represent mean ± SEM. * *p* < 0.05, *** *p* < 0.001, *n* = 3, two-way ANOVA/Tukey’s tests.

**Figure 7:**
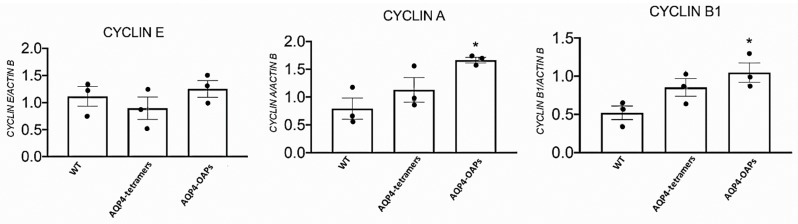
RT PCR expression of the cyclin genes (E, A, and B1) in the U87 cells following M23 AQP4-OAP transfection. * *p* < 0.05 when compared to the experimental group not treated (WT) after 48 h of incubation. • represent the average of each single experiment. Each point represented the mean of at least three experiments± SEM.

Due to the role of KATP channels in proliferation and apoptotic outcomes in gliomas, we tested the hypothesis that Kir6.2-SUR2 and AQP4 collaborate in glioma cell apoptotic fate. To this aim, AQP4-OAPs or AQP4-tetramers expressing glioma cells were treated with zoledronic acid, a Kir6.2-SUR2 K ATP channel inhibitor [45,59], and with KATP channel agonist diazoxide. The expression of AQP4 and F-actin was examined using immunofluorescence (Figure 6A). The results demonstrated that, upon treatment with zoledronic acid, a remarkable change in cell morphology was specifically observed in glioma cells expressing AQP4-OAPs, whereas no such effect was observed in glioma cells expressing AQP4-tetramers. Conversely, the diazoxide treatment did not induce any noticeable effect. More specifically, the application of zoledronic acid transformed AQP4-OAPs expressing U87 cells from irregular-shaped to round-shaped, exhibiting characteristics consistent with apoptosis (Figure 6B). This observation aligns with Coffin’s hypothesis. Notably, the round-shaped cells displayed membrane protrusions, known as “beads-on-a-string”, which are typical apoptotic cell structures previously reported in stressed conditions [21]. Additionally, these cells exhibited membrane blebbing and condensed nuclei. The quantitative analysis (Figure 6B) further confirmed a significantly higher occurrence of round-shaped cells in glioma cells expressing human AQP4-OAPs following treatment with zoledronic acid, as compared to untreated cells. By analyzing nuclear condensation and DNA fragmentation, we observed a SUR2A-specific enhancement of zoledronic-acid-induced apoptosis (Figure 6C). These findings suggest that both AQP4 and Kir6.2 are involved in glioma apoptotic fate. The RT PCR expression of the cyclin genes (E, A, and B1) revealed that M23 AQP4-OAP transfection segregates the cells in the mitotic (M) and G2 states of the cycle. A tendency to the segregation of the U87 cells in the mitotic (M) and G2 states is observed also in the M1 AQP4-tetramer-transfected cells (Figure 7). Therefore, an abnormal enhancement of the functional/expression of the hslo/BK and Kir6.2-SUR2-1/KATP channel subunits were recorded in the AQP4-OAP-transfected U087 cells that were repolarized and were associated with G2-M cycle in comparison to the not transfected cells. The AQP4 tetramer-transfected cells showed reduced KATP and BK channel currents, the downregulation of the hslo/BK channel subunit sensing the intracellular Ca^2+^ ions, thereby favoring the influx of the Ca^2+^ ions possibly carried by TRPV1 with cell depolarization.

## 4. Discussion

In the present work, we showed that the AQP4 aggregation into the pathogenic AQP4-tetramer and AQP4-OAPs differently affected the expression profile of *KCNMA1*, *KCNJ11*, *ABCC8*, and *ABCC9* genes but not of the *KCNJ8* gene in the U87 glioma cell, evaluated by the RTPCR gene expression. In parallel, the endogenous whole cell currents, sustained by the BK and KATP channel subunits and recorded using patch-clamp experiments, were also affected on the same cells. In particular, the expression of the *KCNMA1* gene, encoding for the hSlo pore forming subunit of the BK channels, increased by four-fold in the AQP4-OAP cells, and the currents were enhanced by eight-fold in the physiological range of the membrane potentials of −20 mV (Vm) and −10 mV (Vm), at which BK channels are operative vs. not transfected cells. Conversely, the *KCNMA1* expression decreased by one-fold in the AQP4-tetramer cells in line with the reduced K^+^ ion currents observed in these cells. These data suggest a significant role of the BK channels in mediating the efflux of K^+^ ion associated with AQP4 water efflux in U87 cells. Also, the expression of the *KCNJ11* gene encoding for the pore forming subunit Kir6.2 enhanced by 2.5-fold in the AQP4-OAP cells vs. not transfected cells was observed. The combined expression change of the *KCNMA1* and *KCNJ11* (about 6.5 folds) genes is in the same order of magnitude as the observed enhancement of the K^+^ ion whole cell currents in the AQP4-OAP cells at the physiological range of the membrane potentials of −20 mV and −10 mV (Vm) (about eight-fold), at which these ion channels are operative, supporting the significant contribution of either KATP and BK channels to the K^+^ ion currents in the AQP4-OAP cells.

According to the large K^+^ ion currents recorded in the AQP4-OAP cells, these cells were significantly more repolarized vs. not transfected and AQP4-tetramer-transfected U87 cells. Cell depolarization is indeed associated with mitosis in the cell cycle [58,60,61]. All U87 cells investigated here were depolarized vs. not cancer cells. The AQP4-OAP cells show a reduced volume and high density of ER, and apoptotic cell death vs. the other cells, suggesting a relevant contribution of the BK and KATP channels and AQP4 to this phenomenon. The observed enhanced functional/expression of the KATP and BK channel subunits lead to cell repolarization in the AQP4-OAP cells vs. the not transfected and M1 AQP4-tetramer cells, and the AQP4-OAP cells were more likely to segregate in the G2-M state, an indication of an active proliferative condition.

It should be noted that cell proliferation experiments were performed using the MTT assay, which was metabolized at the mitochondrial level, and, as potassium channels are present in the inner mitochondria membrane, their functionality affects cell viability.

The upregulation of KATP channel subunits SUR2, Kir6.2, and Kir6.1 has been recently associated with cancer in CNS and peripheral tissues [55,62]. The upregulation of the *ABCC8*/SUR1 subunit is associated with better prognosis in pancreatic cancers and in some brain tumors like glioma [34,56].

The stimulation of the KATP activity by growth factors, insulin, and the KATP channel opener minoxidil induces cell proliferation and cancer progression in animals and humans [55]. The gain of function (GOF) mutations of the *ABCC8* and *KCNJ8* subunits is associated with Cantu syndrome, which is characterized by cardiovascular phenotypes in human and animals, and cases of pituitary adenoma in some families [63,64]. The inhibition of KATP channel activity, caused by the apoptotic drug and ion channel modulator staurosporine, as well as KATP channel blockers like sulfonylureas or pyruvate kinase antibodies, leads to the induction of apoptosis and exhibits antiproliferative effects. In line with these data, we found here the upregulation of the KATP channel subunits observed in the AQP4-tetramer and AQP4-OAP cells, and the enhancement of the relative ion channel currents supports the idea that KATP channels are involved in cell proliferation in either type of cell.

We observed that inhibiting KATP activity in AQP4-OAPs using zoledronic acid, which targets either Kir6.1-2 or SUR2 subunit-expressing cells, induced a shift in cell shape from irregular to round. This shape change appears to be associated with different stages of apoptosis. This drug is also a modulator of TRPV1, leading to the inhibition of the TRPV1 channel [65]. This mechanism may contribute to the inhibition of an influx of Ca^2+^ ions in U87 cells. In our cells, the TRPV1 is expressed, but the observed currents were largely carried by BK and KATP channels since they were fully inhibited TEA and BaCl_2_. In addition, the possible contribution of other mechanisms such as the inhibition of farnesyl pyrophosphate synthase by zoledronic acid cannot be excluded to date. Zoledronic acid has been shown to act on different target cells, inducing, for instance, the cell proliferation of osteoblasts but reducing osteoclast activity. The pro-survival and pro-differentiation effects of zoledronic acid in cancer and bone cells are observed between concentrations of 10^−9^ M and 10^−6^ M, while the proapoptotic effects at concentrations >10^−5^ M [47,52] were used in our experiments in the present work. This drug is expected to induce an S-phase arrest or G1 in our cells, as reported in different cell cancer models [66,67,68].

We can speculate that Kir6.2 upregulation in AQP4-OAP-expressing cells is helpful to glioma cells, helping them to escape from apoptosis and survive.

The hslo subunit and its splicing isoforms have a role in different pathophysiological conditions. They are well-known regulators of cell proliferation and are targets of drugs affecting cell death. The upregulation of these ion channels in the presence of AQP4 regulating the efflux of K^+^ ions and water instead selectivity drives the U87 cells to a less pathogenic AQP4-OAP cell aggregation. These effects can be due to protein–protein interaction mechanisms or mediated by second messengers.

In the previous work, the influx of Ca^2+^ ions mediated by the hypotonic-induced activation of mechanosensitive channels was found to be a key step for opening both the BK and intermediate Ca^2+^ (IKCa) channels in normal U87-M GBM cells [69]. Here, these phenomena, the reduced reversal potentials of the K^+^ ion currents in our cells far from the equilibrium potentials for K^+^ ions, can be the result of cationic overactive components like the TRPV1 found in our work and the mechanosensitive channels previously described [69] that allow an additional influx of calcium ions into the cells. In addition, the contribution of different sub-cell GMB populations like neutrospheres in the same culture also differed for the BK channel current expression, and their response to inhibitors can contribute to the BK channel currents recorded in our GBM cells [70].

## 5. Conclusions

The BK channels sustained the efflux of the K^+^ ions associated with AQP4-OAP expression in the U87 cells, and the KATP channels sustained the efflux in the M1 AQP4-tetramer and AQP4-OAP cells. The KATP channel antagonist zoledronic acid proved that it could be effective in targeting pathogenic AQP4-tetramer cell phenotypes, inducing early apoptosis.

## Figures and Tables

**Table 1:** Glioma/brain-cancer-related articles of *ABCC8*, *ABCC9*, *KCNJ11*, *KCNJ8*, *AQP4* and *KCNMA1* genes in PubMed.

	Differential Expression/Mutation	Cancers	Information on Analyzed Samples	Consequences	PubMed Code
	Upregulation	Glioma	Tissue from 22 patients with newly diagnosed GBM.		[26,27]
	Upregulation	Glioma	A total of 50 glioma samples, ranging in grades from II to IV, were collected from a cohort of 35 male and 15 female patients.	Cells exhibiting enhanced migration potential displayed notably high levels of AQP4 expression, indicating a potential association between AQP4 and glioma cell migration.	[28]
	Upregulation	Brain tumors	The study involved analyzing 5 tumor samples from subependymomas located in the fourth ventricle, as well as subependymoma (SE) tumor samples found supratentorial with relation to the first to the third ventricle.	Increased AQP4 expression has been observed in malignant tumors, where it appears to contribute to edema formation, invasive growth, and tumor recurrence. However, AQP4 does not play a significant role in benign tumors.	[29]
AQP4	Upregulation	GBM	The tumor samples from 14 patients with primary glioblastomas.		[30]
	Upregulation	Brain tumors/GBM	Tissue samples from brain tumors of 26 patients.	Analysis of GBM samples showed that increased AQP4 expression, loss of cellular polarity, and matrix alterations are linked to more severe glioblastoma and cerebral edema. This highlights AQP4’s role in tumor malignancy, suggesting it as a potential therapeutic target.	[31]
	Upregulation (*AQP4-tetramer* and *AQP4-OAP*)	Brain tumors/GBM	Tumor tissues obtained from a total of 22 patients diagnosed with astrocytoma of WHO grades II, III, and IV, and an additional patient diagnosed with glioblastoma multiforme (GBM), were included in the study.	Upregulation of *AQP4*-tetramers and mRNA-AQP4-OAPs in all astrocytomas, but the *AQP4-OAP’s*/*AQP4-tetramer’s* ratio differed from 1.14 to 1.5 in low-grade astrocytomas and to 1.94 in glioblastomas. This could have a possible impact on the development of new therapies.	[28]
	Upregulation	Brain tumors/GBM	Brain tumors and the corresponding adjacent tissues from 30 patients diagnosed with glioblastoma.	The overexpression of *AQP4* was observed in both brain tumors and the adjacent tissues, and this heightened expression was found to be correlated with the extent of brain edema.	[32]
	Downregulation	Brain tumors/GBM	A total of 16 tissue samples were collected from various regions within the tumoral core.	The presence of AQP4 alterations in GBMs appears to play a role in edema formation. Therefore, AQP4 could be viewed as a promising early biomarker for tracking GBM progression and also as a potential target for AQP4 modulation in therapeutic approaches.	[33]
KATP					
	Upregulation of *ABCC8*	Glioma	The information is based on the analysis of 1893 human glioma samples from four independent databases.	Glioma chemosensitivity can be predicted by high ABCC8 mRNA expression, whereas low ABCC8 mRNA expression can serve as an indicator of glioma sensitivity to radiotherapy.	[34]
	Upregulation of *ABCC8*	Brain tumors	The information comes from the analysis of human tissue samples from 6 glioblastoma, 12 brain metastases, 11 medulloblastoma, 9 supratentorial ependymomas, and 8 posterior fossa ependymomas.	SUR1 is a potential therapeutic target for reducing neuroinflammation in adult and pediatric brain tumors. Inhibition of SUR1 induces neuronal stabilization in glioblastoma, brain metastases, and posterior fossa ependymoma, as well as edema reduction in medulloblastoma.	[35]
	Upregulation of *KCNJ8* and *ABCC8*	Glioma	20 human glioma biopsies.	The Kir6.2 and SUR1 subunits of the KATP channel are involved in the proliferation of U87 and U251 glioma cells. The KATP channel inhibitors significantly reduced the growth curve. On the other hand, KATP channel agonists promoted the proliferation of U87 and U251 cells.	[36]
BK	Upregulation of *KCNMA1*	Glioma	Biopsies from patients with malignant gliomas.	The expression of BK channels has shown a positive correlation with tumor malignancy grades, indicating a significant role for the gBK channel in glioma biology. Utilizing BK channel agonists could potentially be advantageous for brain tumor patients, as they might enhance the delivery of anti-neoplastic agents to brain tumors.	[37]
	Upregulation of *KCNMA1*	Brain tumors	Sample tissues from patients with malignant gliomas.		[38]

## Data Availability

The data are available from the authors on request.
